# Development of CpG-adjuvanted stable prefusion SARS-CoV-2 spike antigen as a subunit vaccine against COVID-19

**DOI:** 10.1038/s41598-020-77077-z

**Published:** 2020-11-18

**Authors:** Tsun-Yung Kuo, Meei-Yun Lin, Robert L. Coffman, John D. Campbell, Paula Traquina, Yi-Jiun Lin, Luke Tzu-Chi Liu, Jinyi Cheng, Yu-Chi Wu, Chung-Chin Wu, Wei-Hsuan Tang, Chung-Guei Huang, Kuo-Chien Tsao, Charles Chen

**Affiliations:** 1 Medigen Vaccine Biologics Corporation, Taipei, Taiwan; 2 Department of Biotechnology and Animal Science, National Ilan University, Yilan City, Yilan County Taiwan; 3 Dynavax Technologies, Emeryville, CA 94608 USA; 4 Department of Laboratory Medicine, Linkou Chang Gung Memorial Hospital, Taoyuan City, Taiwan; 5 Research Center for Emerging Viral Infections, College of Medicine, Chang Gung University, Taoyuan City, Taiwan; 6 College of Science and Technology, Temple University, Philadelphia, PA 19122 USA

**Keywords:** Immunology, Microbiology

## Abstract

The COVID-19 pandemic is a worldwide health emergency which calls for an unprecedented race for vaccines and treatment. In developing a COVID-19 vaccine, we applied technology previously used for MERS-CoV to produce a prefusion-stabilized SARS-CoV-2 spike protein, S-2P. To enhance immunogenicity and mitigate the potential vaccine-induced immunopathology, CpG 1018, a Th1-biasing synthetic toll-like receptor 9 (TLR9) agonist was selected as an adjuvant candidate. S-2P in combination with CpG 1018 and aluminum hydroxide (alum) was found to be the most potent immunogen and induced high titer of neutralizing antibodies in sera of immunized mice against pseudotyped lentivirus reporter or live wild-type SARS-CoV-2. In addition, the antibodies elicited were able to cross-neutralize pseudovirus containing the spike protein of the D614G variant, indicating the potential for broad spectrum protection. A marked Th1 dominant response was noted from cytokines secreted by splenocytes of mice immunized with CpG 1018 and alum. No vaccine-related serious adverse effects were found in the dose-ranging study in rats administered single- or two-dose regimens of S-2P combined with CpG 1018 alone or CpG 1018 with alum. These data support continued development of CHO-derived S-2P formulated with CpG 1018 and alum as a candidate vaccine to prevent COVID-19 disease.

## Introduction

COVID-19 was first identified as a cause of severe pneumonia cases in December 2019 in association with a seafood market in Wuhan, China^1^. The viral agent was identified as a novel SARS-like coronavirus (SARS-CoV-2) most closely related to bat coronavirus^1^. In the 10 months since its first appearance, SARS-CoV-2 has become the largest pandemic since the 1918 influenza with over 50 million infected and over 1.2 million deaths worldwide as of November 2020^2,3^. The rapid spread and huge socioeconomic impact of this pandemic require the urgent development of effective countermeasures, including vaccines. In response, pharmaceuticals, academia, and institutions are developing vaccines and drugs at an unprecedented pace with governments and foundations pledging hundreds of millions of dollars for COVID-19 research^4^.

In addition to basic public health control measures such as social distancing, contact tracing and quarantine, a safe and effective vaccine is the only weapon that can potentially offer lasting protection against COVID-19 and stop the current pandemic. According to the WHO, 26 vaccine candidates using various platforms have entered clinical trials as of July 31, 2020^5^. These candidate vaccines have all been developed in compliance with WHO guidelines that define desired characteristics such as dose regimen, target population, safety, measures of efficacy, and requirements for product stability and storage^6^.

Coronaviruses are among the largest known enveloped RNA viruses and cause respiratory illnesses in humans ranging from the common cold to SARS, MERS, as well as the current COVID-19 pandemic^7^. Similar to SARS-CoV, the spike (S) protein of SARS-CoV-2 is the receptor for attachment and cell entry via the cellular receptor hACE2^1^. Researchers are also adapting antigen design strategies used for SARS-CoV and MERS-CoV spike proteins to develop a candidate vaccine for SARS-CoV-2. A stabilized prefusion form of the MERS spike protein was achieved in 2017 by transferring two proline substitutions between heptad repeat 1 and the central helix analogous to those defined in the HKU1 spike protein. These mutations together with a C-terminus foldon trimerization domain stabilized the spike ectodomain in its prefusion state resulting in a more potent immunogen with dose-sparing properties compared to protein made with the original wild-type sequence^8^. The analogous mutations in the SARS-CoV-2 spike resulted in a homogeneous population of proteins allowing the atomic-level structure to be solved by cryo-electron microscopy^9^.

Subunit vaccines such as the spike protein are often poorly immunogenic by themselves and therefore typically require adjuvants to enhance their ability to produce an immune response^10^. Adjuvants can be classified based on their properties into several categories, including aluminum salt-based (aluminum hydroxide and aluminum phosphate), oil emulsion-based such MF59 and AS03 (both squalene-based), and toll-like receptor (TLR) agonists, including TLR9 agonist CpG 1018, and TLR4 agonist monophosphoryl lipid A (MPL)-based AS01B (MPL and saponin QS-21) and AS04 (MPL and aluminum hydroxide)^11^. A previous study employing four different candidate vaccines against SARS-CoV, all of them with and without aluminum-based adjuvants successfully elicited neutralizing antibodies and conferred protection against infection; however, tissue damage due to immunopathology was also observed from infiltrating lymphocytes^12^. Historical evidence from animal models suggests that vaccine-primed Th2 and Th17 responses may be associated with immunopathology referred to as vaccine-associated enhanced respiratory disease (VAERD)^13^; therefore, extra caution must be taken in the choice of adjuvants. Synthetic oligodeoxynucleotides with CpG motifs (CpG ODN) are agonists for TLR9 and mimic the activity of naturally occurring CpG motifs found in bacterial DNA. CpG ODN are potent vaccine adjuvants generating Th1-biased responses, thus making them a good choice to mitigate potential Th2 and Th17 induced immunopathology^11^. CpG 1018, a 22-mer CpG ODN containing sequence with a modified phosphorothioate backbone^14^, is the adjuvant used in the licensed hepatitis B vaccine HEPLISAV-B, and is the only TLR9 agonist used in an approved vaccine.

In this study, we present data from preclinical studies aimed at developing a COVID-19 candidate subunit vaccine using CHO cell-expressed SARS-CoV-2 S-2P antigen combined with various adjuvants. We have shown that S-2P, when mixed with CpG 1018 and aluminum hydroxide adjuvants, was most effective in inducing antibodies that neutralized pseudovirus and wild-type live virus while minimizing Th2-biased responses with no vaccine-related adverse effects.

## Results

### Induction of potent neutralizing antibodies by CpG 1018 and aluminum hydroxide-adjuvanted S-2P

**Figure 1:**
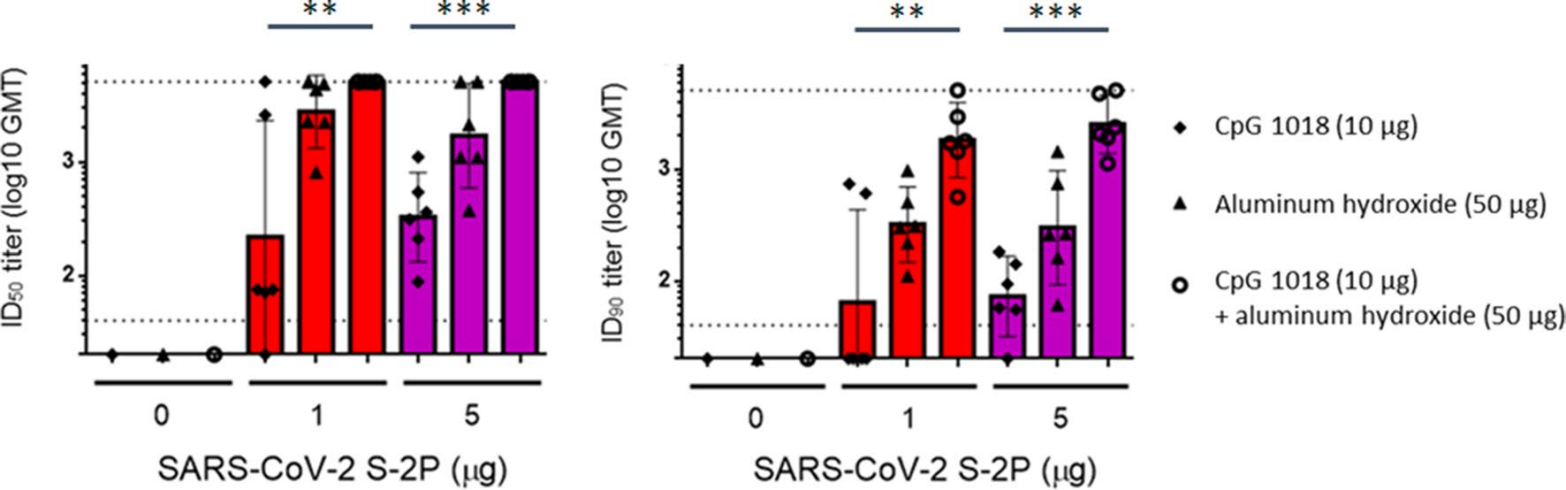
Induction of neutralizing antibodies by CpG 1018 and aluminum hydroxide-adjuvanted SARS-CoV-2 S-2P 2 weeks post-second injection. BALB/c mice (N = 6 per group) were immunized with 2 dose levels of CHO cell-expressed SARS-CoV-2 S-2P adjuvanted with CpG 1018, aluminum hydroxide or a combination of both 3 weeks apart and the antisera were harvested at 2 weeks after the second injection. The antisera were subjected to neutralization assay with pseudovirus expressing SARS-CoV-2 spike protein to determine the ID_50_ (left) and ID_90_ (right) titers of neutralization antibodies.

**Figure 2:**
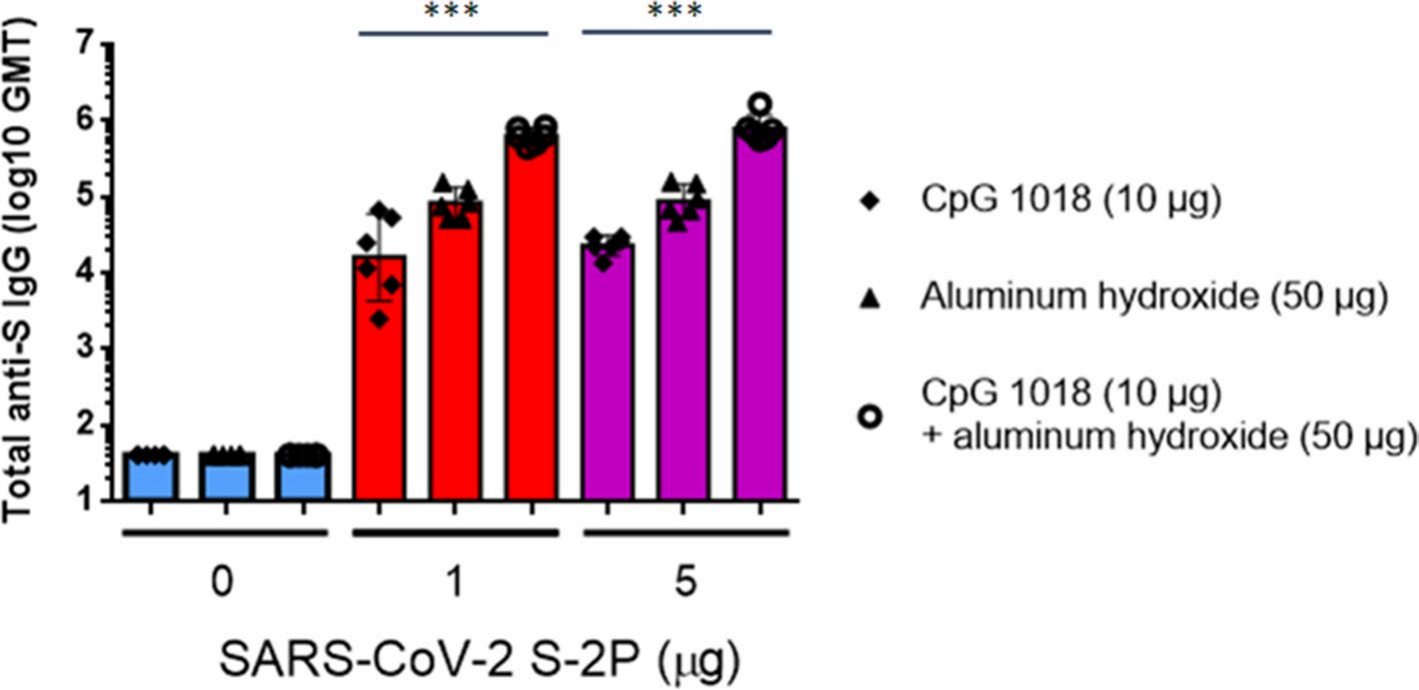
Total anti-S IgG titers in mice immunized with S-2P with adjuvants**.** Sera from BALB/c mice in Fig. 1 (N = 6 per group) immunized with 0, 1 or 5 μg of S-2P with CpG 1018, aluminum hydroxide or combination of both were quantified for the total amount of anti-S IgG with ELISA.

**Figure 3:**
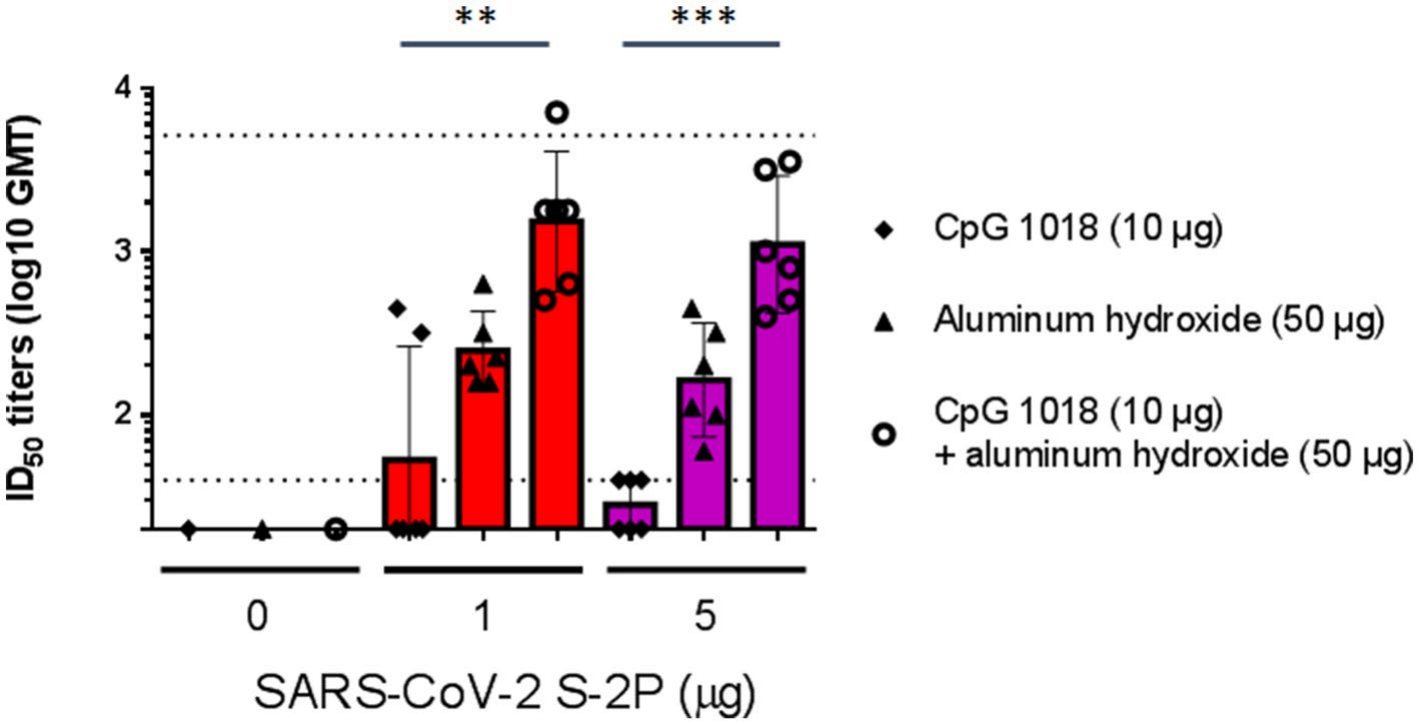
Neutralization of wild-type SARS-CoV-2 virus by antibodies induced by SARS-CoV-2 S-2P adjuvanted with CpG 1018 and aluminum hydroxide. The antisera were collected as described in Fig. 2 (N = 6 per group) and subjected to a neutralization assay with wild-type SARS-CoV-2 to determine neutralization antibody titers.

**Figure 4:**
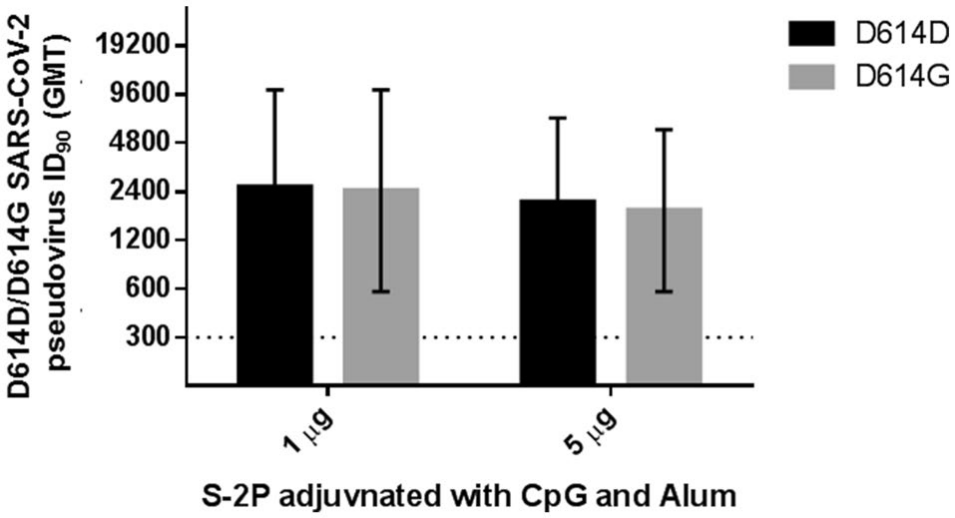
Inhibition of pseudoviruses carrying D614D (wild-type) or D614G (variant) versions of the spike protein by mice immunized with S-2P with CpG 1018 and aluminum hydroxide. The antisera of BALB/c mice immunized with 1 or 5 μg of S-2P with 10 μg CpG 1018 and 50 μg aluminum hydroxide as in Fig. 1 (N = 5 per group due to assay capacity) were collected. Neutralization assays were performed with pseudoviruses with either D616D or D614G spike proteins.

**Figure 5:**
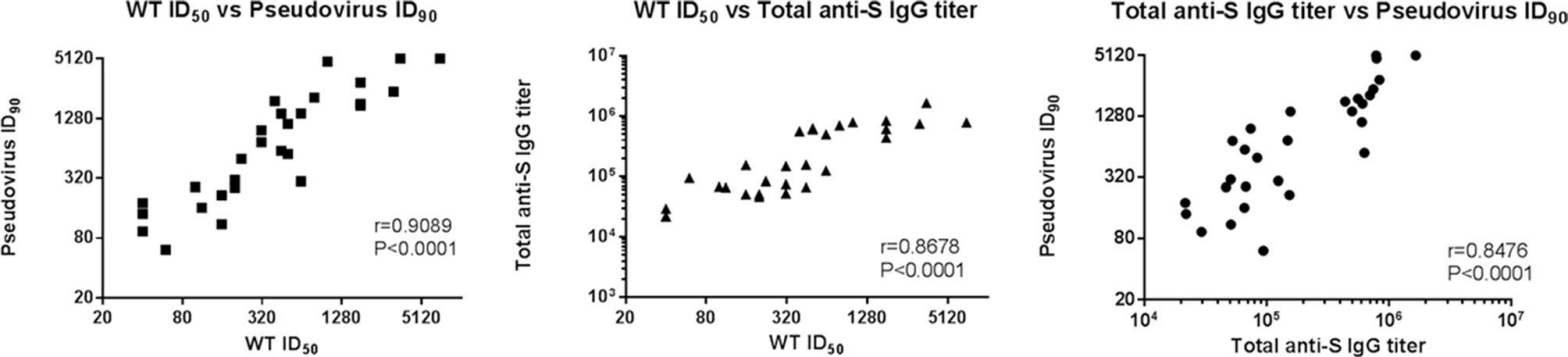
Correlations between SARS-CoV-2 pseudovirus ID_90,_ wild-type SARS-CoV-2 ID_50_, and total anti-S IgG titers in mice. Values of ID_50_/ID_90_/IgG titers above lower detection limit (> 40) (N = 29) were tabulated and correlations were calculated with Spearman’s rank correlation coefficient for wild-type SARS-CoV-2 ID_50_ vs pseudovirus ID_90_ (left), wild-type SARS-CoV-2 ID_50_ vs total anti-S IgG titer (middle), and pseudovirus ID_90_ vs total anti-S IgG titer (right).

To facilitate establishment of stable clones for clinical studies and commercial production, we applied the ExpiCHO system as the expression system of S-2P antigen. The S-2P proteins produced in CHO cells and their structure displayed typical spike trimers under cryo-EM (Supplementary Figure S1), resembling that of 293-expressed SARS-CoV-2 S protein^9^, suggesting that CHO cells are feasible in production of S-2P. We next examined the potential of Th1-biasing CpG 1018 for clinical use. Aluminum hydroxide (hereafter abbreviated as alum) was tested along with CpG 1018 since alum has been characterized to enhance the potency of CpG adjuvant when used in combination while also retaining the property of inducing Th1 responses^15^. The pseudovirus neutralization assay was performed with sera drawn either 3 weeks after the first injection or 2 weeks after the second injection. At 3 weeks after the first injection, neutralizing activities were already observed when mice were immunized with both 1 and 5 µg of S-2P with CpG 1018 and alum (Supplementary Figure S2). At 2 weeks after the second injection, reciprocal inhibition dilution 50 (ID_50_) GMT of 245, 3109, and 5120 were obtained with immunization of 1 µg S-2P adjuvanted with CpG 1018, alum, and with both CpG 1018 and alum, respectively (Fig. 1). Similar trends were observed at 5 µg of S-2P in both BALB/c and C57BL/6 mice (Figs. 1 and S3). Sera from these mice were then examined for the amount of anti-S IgG. CpG 1018 in combination with alum produced significantly higher titers of anti-S IgG compared to CpG 1018 alone (Figs. 2 and S4). To confirm the activities of the antibodies against the critical receptor-binding domain (RBD) of the S protein, immune sera were examined for anti-RBD IgG and the results were similar to that of the anti-S IgG with S-2P in combination with both CpG 1018 and alum induced the highest amount of IgG titer (Supplementary Figure S5). There was a moderate correlation between anti-S IgG and anti-RBD IgG titers as shown by Spearman’s rank correlation coefficient of 0.6486 (Supplementary Figure S6). The immune sera were further tested for their neutralization capabilities against wild-type SARS-CoV-2 in a neutralization assay. S-2P was able to inhibit SARS-CoV-2 at a concentration of 1 µg, although at lower potency than that of pseudovirus (Figs. 1 and 3). The reciprocal ID_50_ GMT of 1 µg S-2P in the presence of CpG 1018, alum, and with both CpG 1018 and alum were approximately 60, 250, and 1500, respectively (Fig. 3). Pseudovirus carrying the current dominant D614G variant spike was also generated and neutralizing antibodies from mice immunized with S-2P with CpG 1018 and alum were effective against both pseudoviruses carrying the wild-type D614 and mutant D614G versions of spike proteins (Fig. 4). Neutralization titers of wild-type virus and pseudovirus and total anti-S IgG titers were all found to be highly correlated with Spearman’s rank correlation coefficients greater than 0.8 (Fig. 5).

### CpG 1018 induced Th1 immunity

**Figure 6:**
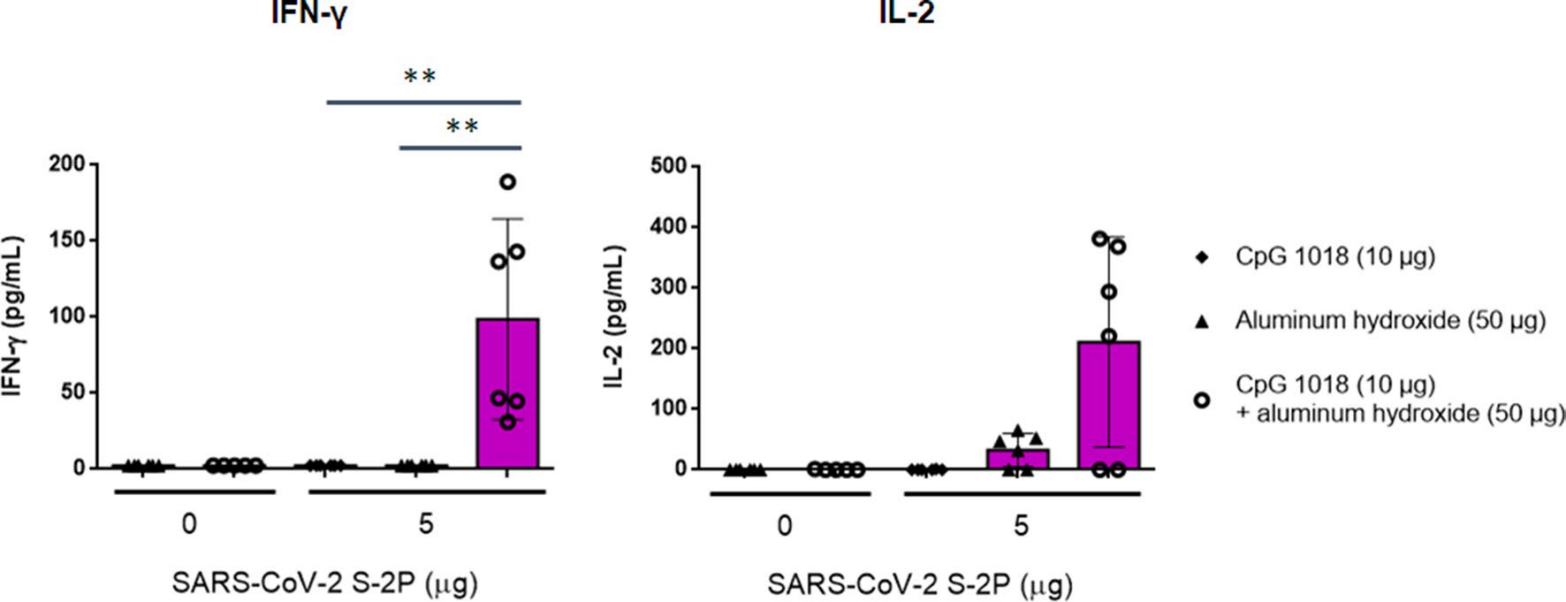
Th1-dependent cytokine production induced by SARS-CoV-2 S-2P adjuvanted with CpG 1018, aluminum hydroxide, or CpG 1018 with aluminum hydroxide in mice. The antisera of BALB/c mice (N = 6 per group) were collected at 2 weeks after the second injection, the splenocytes were harvested and incubated with S-2P protein (2 μg), concanavalin A (0.1 μg; data not shown) for positive control, or complete RPMI 1640 medium only for negative control. After 20 h incubation, the levels of IFN-γ (left) and IL-2 (right) were analyzed by ELISA.

**Figure 7:**
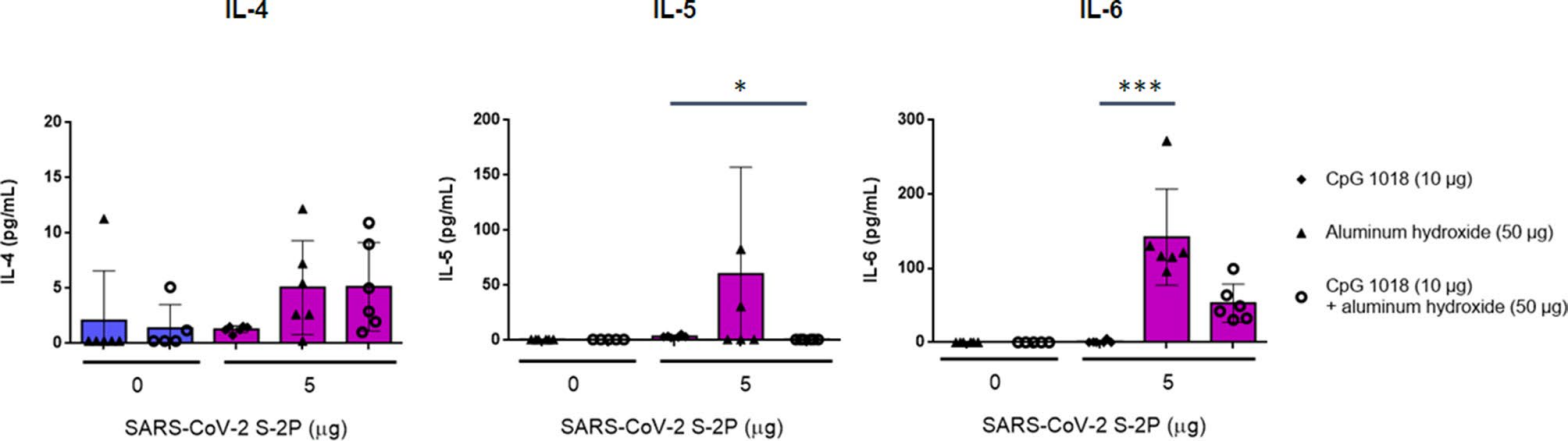
Th2-dependent cytokine production induced by SARS-CoV-2 S-2P adjuvanted with CpG 1018, aluminum hydroxide, or CpG 1018/aluminum hydroxide in mice. The antisera of BALB/c mice (N = 6 per group) were collected at 2 weeks after the second injection, the splenocytes were harvested and stimulated as in Fig. 6. After 20 h incubation, the levels of IL-4 (left), IL-5 (middle), and IL-6 (right) released from the splenocytes were analyzed. For detection of cytokines, the culture supernatant was harvested to analyze the levels of cytokines by ELISA.

**Figure 8:**
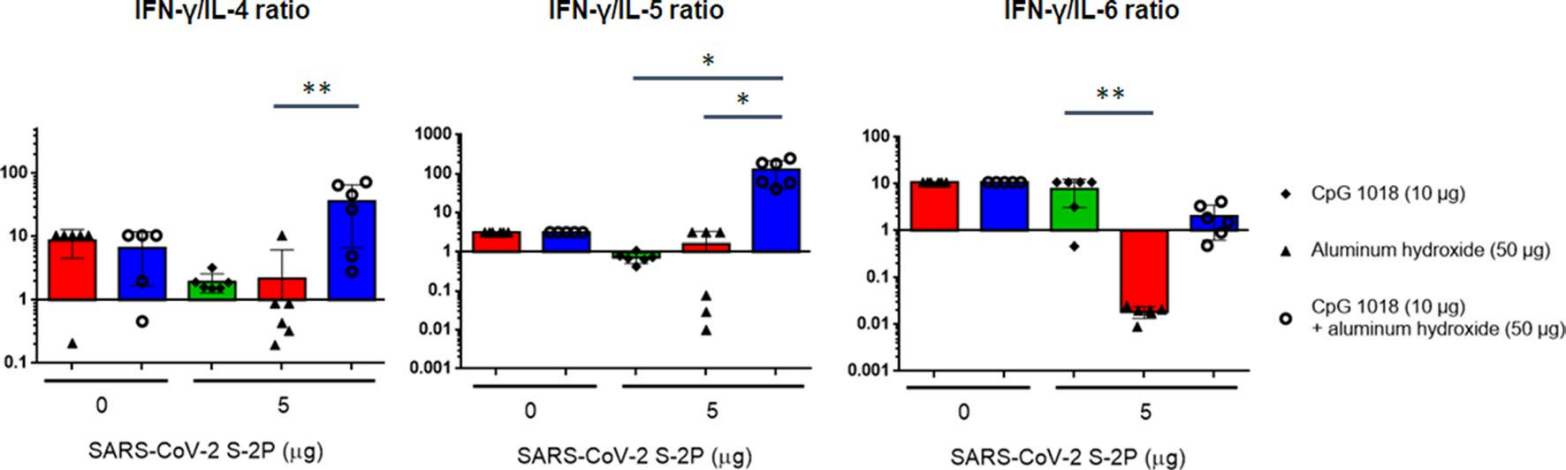
IFN-γ/IL-4, IFN-γ/IL-5, and IFN-γ/IL-6 ratios. IFN-γ, IL-4, IL-5, and IL-6 values from the cytokine assays (N = 6 per group) were used to calculate ratios. Ratio values greater than 1 indicate Th1 bias whereas ratio less than 1 indicate Th2 bias responses.

To identify whether CpG 1018 could induce Th1 responses in our vaccine-adjuvant system, cytokines involved in Th1 and Th2 responses were measured in splenocytes from mice immunized with S-2P with alum, CpG 1018, or combination of the two. As expected, S-2P adjuvanted with alum induced limited amounts of IFN-γ and IL-2, the representative cytokines of Th1 response. In contrast, significant increases in IFN-γ and IL-2 were detected most strongly in high antigen dose plus CpG 1018 and alum (Fig. 6). For Th2 response, while the levels of IL-4, IL-5 and IL-6 increased in the presence of alum and S-2P, addition of CpG 1018 to alum suppressed the levels of IL-5 and IL-6 (Fig. 7). IFN-γ/IL-4, IFN-γ/IL-5, and IFN-γ/IL-6 ratios are strongly indicative of a Th1-biased response and were increased by approximately 36-, 130-, and twofold, respectively, in the presence of S-2P combined with CpG 1018 and alum (Fig. 8). These results suggested that the effect of CpG 1018 is dominant over alum in directing the cell-mediated response towards Th1 response, while retaining high antibody levels.

### S-2P did not result in systemic adverse effects in rats

To elucidate the safety and potential toxicity of the vaccine candidate, 5 μg, 25 μg or 50 μg of S-2P adjuvanted with 1500 μg CpG 1018 or 750 μg CpG 1018 combined with 375 μg alum were administered to SD rats for single-dose and repeat-dose studies. No mortality, abnormality of clinical signs, differences in body weight changes, body temperature, nor food consumption were observed in either gender that could be attributed to S-2P (with or without adjuvant) with single dose administration (Supplementary Figures S7 and S8). Increased body temperature at 4-h or 24-h after dosing was found in both genders of single-dose study and repeat-dose study; however, these temperature changes were moderate and were recovered after 48-h in both genders of all treated groups including controls (PBS) (Supplementary Figure S8). No gross lesions were observed in organs of most of the male and female rats with single-dose and two-dose administration, except for one male rat which was deemed to be non-vaccine-related. In conclusion, S-2P protein, with CpG 1018 or CpG 1018 with alum as adjuvants administrated intramuscularly once or twice to SD rats did not induce any systemic adverse effect.

## Discussion

In this study, we showed that in mice, two injections of a subunit vaccine consisting of the prefusion spike protein (S-2P) adjuvanted with CpG 1018 and alum were effective in inducing potent neutralization activity against both pseudovirus expressing wild-type and D614G variant spike proteins, and wild-type SARS-CoV-2. The combination of S-2P with CpG 1018 and alum elicited Th1-dominant immune responses with high neutralizing antibody levels in mice and showed no major adverse effects in rats. We also successfully scaled-up yield of S-2P by establishing stable CHO cell clones expressing S-2P protein and improved the purification process at a sufficient quantity of antigen for the production of a commercial vaccine. Spike is a highly glycosylated protein and we chose CHO-cell production to achieve mammalian glycosylation patterns that will include complex glycans and may be important for immunogenicity. Although the leading subunit protein COVID-19 vaccines by developers such as Sanofi Pasteur and Novavax are made in baculovirus, the insect cells produce protein with man-9 glycosylation that may be sufficient for immune response induction, but may not recapitulate the antigenicity of virus grown in mammalian cells^5,16,17^. Animal challenge studies will be conducted at a future date to examine the safety and efficacy of our candidate vaccine. Based on our results and in accordance with the International Coalition of Medicines Regulatory Authorities (ICMRA), we plan to move forward with first-in-human clinical trials and conduct preclinical studies in parallel to expedite vaccine development in the current COVID-19 pandemic.

We have successfully shown robust immunogenicity elicited by adjuvanted SARS-CoV-2 S-2P (Figs. 1 and 2). Much stronger neutralizing antibody responses were detected in mice when 1 μg or 5 μg of S-2P protein was adjuvanted with 10 μg of CpG 1018 and 50 μg of alum than with either adjuvant alone (Fig. 1). S-2P in conjunction with CpG 1018 and alum induced potent anti-S antibodies that were effective against wild-type virus (Figs. 2 and 3). As RBD region is the key for receptor attachment and cellular entry of SARS-CoV-2, we have shown that the anti-S antibodies elicited by S-2P and adjuvants were also able to bind to RBD (Supplementary Figure S5), which is unsurprising since RBD contains key epitopes for neutralizing antibody binding and may be useful for screening potent neutralizing antibodies^18^. We have also noticed that the individual mice anti-RBD IgG titers varied more widely than that of anti-S IgG titers (Fig. 2 and S5). This may have been due to the polyclonal nature of the anti-S antibodies, in that anti-RBD antibodies are only a subset of anti-S antibodies and other anti-S antibodies target other regions of the S protein. We have shown that high degrees of correlation between neutralization titers of pseudovirus, wild-type virus, and anti-S IgG titers (Fig. 5). Therefore, anti-S IgG titers could be used as indicators of immunogenicity during the production and quality control process. Additionally, anti-S and anti-RBD IgG titers showed a moderate correlation between the two (Supplementary Figure S6), which suggested that while not all anti-S antibodies were directed at the RBD region, the anti-S antibodies were still effective in neutralizing activities. During the course of vaccine development against fast-evolving RNA viruses such as SARS-CoV-2, it is important that the protection offered by the vaccine could extend to variants that could otherwise drastically reduce the effectiveness of neutralizing antibody. Strains harboring the D614G mutation in the spike protein were first observed in Europe in February 2020 and over time has become the global dominant variant^19^. Our results of the pseudovirus neutralization assay showed cross-reaction of these antibodies with the dominant circulating strain D614G with similar titer levels (Fig. 4). Therefore, we confirmed that S-2P was able to generate antibodies effective against both the original wild-type strain and its variant. Neutralization titers of antibodies against different strains of wild-type viruses should be investigated in the future, but our results indicate the potential of this candidate vaccine to provide broad spectrum protection against COVID-19 infection.

Although moderate IL-4 production was detected in mice receiving 5 μg of S-2P combined with CpG 1018 and alum, the IFN-γ/IL-4 ratio was 16-fold higher than those receiving 5 μg of S-2P adjuvanted with alum alone. These results suggested that CpG 1018, even in the presence of alum could steer the immune response away from Th2 to a Th1 response. Moreover, these mice produce a limited amount of IL-5, which is a key mediator in eosinophil activation and major regulator of eosinophil accumulation in tissues^20^. Previous studies showed that the lung-infiltrating eosinophils were a common indication of Th2-biased immune responses seen in animal models testing SARS-CoV vaccine candidates^12^. The finding that IL-5 production was inhibited by the S-2P adjuvanted with CpG 1018 plus alum suggests that it would be less likely to induce immune responses resulting in eosinophil infiltration in lung. Th1- and Th2-biased responses are determined by factors, including administration routes, antigen and adjuvant characteristics, and cytokines^11^. Our results showed that S-2P per se is unlikely to skew the immune response towards Th1, but in the presence of an adjuvant such as CpG 1018, S-2P can direct the immune response towards Th1. Thus, we have shown that S-2P adjuvanted with CpG 1018 plus alum is a potential formulation for COVID-19 vaccine development.

CpG 1018 is currently the only FDA-approved CpG adjuvant for human vaccine use^11^. While CpG 1018 without alum proved to be an effective adjuvant in the hepatitis B vaccine HEPLISAV-B^21^, the current study involving S-2P pointed out that CpG 1018 alone performed relatively poorly in neutralizing antibody induction (Figs. 1 and 3). The mechanism of this difference is unclear, but may be due to the different nature of the antigens (hepatitis B surface antigen HBsAg vs SARS-CoV-2 prefusion spike protein S-2P) and the fact that SARS-CoV-2 is a novel pathogen and many of its peculiarities have yet to be understood. We have observed that CpG 1018 itself is a fairly effective adjuvant in mice, but is substantially enhanced by the addition of alum [unpublished]. In the studies leading to the approval of HEPLISAV-B, very high rates of seroprotection in older age groups have been demonstrated while Engerix-B, another hepatitis B vaccine which utilizes alum as an adjuvant shows a pronounced loss of vaccine efficacy in age groups over 40 in these studies^14^. A recent study comparing both hepatitis B vaccines in a geriatric population aged 60–70 years with diabetes mellitus was able to demonstrate higher level of seroprotection and immunogenicity in CpG 1018-adjuvanted vaccine than alum-adjuvanted vaccine^22^. Therefore, we believe that a COVID-19 vaccine containing CpG 1018 will be particularly well suited for older adults and adults with certain forms of immunocompromise, such as diabetes and chronic kidney disease. However, there is at present very little data on efficacy or safety of CpG 1018 in vaccines in pediatric populations and these questions remain to be answered in future studies. As of our current data, the COVID-19 vaccine would be targeted at a population of 20 to 70 years of age.

Single-dose or repeat-dose administration of S-2P protein adjuvanted with 750 μg CpG 1018 and 375 μg alum was well tolerated in rats in both genders, supporting human clinical trials in young healthy adults. GLP toxicology study of S-2P in combination of higher dose of CpG 1018 and alum will be conducted to explore safety of the formulated S-2P for dose escalation study in human clinical trials with the elderly and those with chronic health conditions such as diabetes and cardiovascular diseases, who may require a higher adjuvant dose to boost the immune systems. As a two-dose regimen of S-2P formulated with CpG 1018 and alum induced potent neutralizing activity, our future plans will include testing single-dose regimens.

Our study showed that CHO-derived S-2P proteins elicited robust immune responses in mice, indicating that CHO cell is an appropriate platform for stable S-2P production in vaccine development. Other vaccines using CHO cells to produce antigens include hepatitis B vaccines GenHevacB and Sci-B-Vac^23^. To this date, we have established stable CHO cell clones expressing S-2P and the one with the highest yield will be selected to produce master cell bank for large scale GMP production of commercial vaccine.

The rapid spread of SARS-CoV-2 and urgent need for an effective vaccine call for its development using readily available and proven technologies. The spike protein is the main receptor binding and membrane fusion protein, which serves as the major antigen target for COVID-19 vaccine development. We have demonstrated in this study that the S-2P combined with the advanced adjuvant CpG 1018, the adjuvant contained in the FDA-approved adult hepatitis B vaccine (HEPLISAV-B), in combination with alum induced potent Th1-biased immune responses to prevent wild-type virus infections while retaining high antibody levels that show cross-neutralization of variant viruses. Therefore, this vaccine formulation serves as an ideal vaccine candidate in alleviating the burden of the global COVID-19 pandemic.

## Methods

### Production of S-2P protein ectodomains from ExpiCHO-S cells

The plasmid expressing SARS-CoV-2 (strain Wuhan-Hu-1 GenBank: MN908947) S protein ectodomain was obtained from Dr. Barney S. Graham (Vaccine Research Center, National Institute of Allergy and Infectious Diseases, USA) and contains a mammalian-codon-optimized gene encoding SARS-CoV-2 S residues 1–1208 with a C-terminal T4 fibritin trimerization domain, an HRV3C cleavage site, an 8 × His-tag and a Twin-Strep-tag ^9^. The S-2P form was created by mutation of the S1/S2 furin-recognition site 682-RRAR-685 to GSAS to produce a single-chain S0 protein, and the 986-kV-987 was mutated to PP^9^.

ExpiCHO-S cells (Thermo Fisher Scientific) were transfected with the plasmid expressing S-2P protein ectodomains by ExpiFectamine CHO transfection kit (Thermo Fisher Scientific), respectively. The secreted S-2P protein was purified by affinity chromatography. Purification tags were removed by HRV3C protease digestion and the S-2P protein was further purified. The purified S-2P proteins produced from ExpiCHO-S cells were quantified by BCA assay (Thermo Fisher Scientific), flash frozen in liquid nitrogen and then stored at − 80 °C. The ExpiCHO-expressed S-2P was sent to the Electronic Microscopy Laboratory at the Advanced Technology Research Facility (National Cancer Institute) for cryo-EM confirmation.

### Pseudovirus production and titration

To produce SARS-CoV-2 pseudoviruses, a plasmid expressing full-length wild-type Wuhan-Hu-1 strain SARS-CoV-2 spike protein was cotransfected into HEK293T cells with packaging and reporter plasmids pCMVΔ8.91 and pLAS2w.FLuc.Ppuro (RNAi Core, Academia Sinica), using TransIT-LT1 transfection reagent (Mirus Bio). Site-directed mutagenesis was used to generate the D614G variant by changing nucleotide at position 23403 (Wuhan-Hu-1 reference strain) from A to G. Mock pseudoviruses were produced by omitting the p2019-nCoV spike (WT). Seventy-two hours post-transfection, supernatants were collected, filtered, and frozen at − 80 °C. The transduction unit (TU) of SARS-CoV-2 pseudotyped lentivirus was estimated by using cell viability assay in response to the limited dilution of lentivirus. In brief, HEK-293 T cells stably expressing human ACE2 gene were plated on 96-well plate 1 day before lentivirus transduction. For the titering of pseudovirus, different amounts of pseudovirus were added into the culture medium containing polybrene. Spin infection was carried out at 1100×*g* in 96-well plate for 30 min at 37 °C. After incubating cells at 37 °C for 16 h, the culture media containing virus and polybrene were removed and replaced with fresh complete DMEM containing 2.5 μg/ml puromycin. After treating with puromycin for 48 h, the culture media were removed and cell viability was detected by using 10% AlarmaBlue reagents according to manufacturer’s instruction. The survival rate of uninfected cells (without puromycin treatment) was set as 100%. The virus titer (transduction units) was determined by plotting the survival cells versus diluted viral dose.

### Pseudovirus-based neutralization assay

HEK293-hAce2 cells (2 × 10^4^ cells/well) were seeded in 96-well white isoplates and incubated for overnight. Sera were heated at 56 °C for 30 min to inactivate complement and diluted in MEM supplemented with 2% FBS at an initial dilution factor of 20, and then twofold serial dilutions were carried out (for a total of 8 dilution steps to a final dilution of 1:5120). The diluted sera were mixed with an equal volume of pseudovirus (1000 TU) and incubated at 37 °C for 1 h before adding to the plates with cells. After the 1 h incubation, the culture medium was replaced with 50 μL of fresh medium. On the following day, the culture medium was replaced with 100 μL of fresh medium. Cells were lysed at 72 h post infections and relative luciferase units (RLU) were measured. The luciferase activity was detected by Tecan i-control (Infinite 500). The 50% and 90% inhibition dilution titers (ID_50_ and ID_90_) were calculated considering uninfected cells as 100% neutralization and cells transduced with only virus as 0% neutralization. Reciprocal ID_50_ and ID_90_ geometric mean titers (GMT) were both determined as ID_90_ titers are useful when ID_50_ titer levels are consistently saturating at the upper limit of detection.

### Wild-type SARS-CoV-2 neutralization assay

The neutralization assay with SARS-CoV-2 virus was conducted as previously reported^24^. Vero E6 cells (2.5 × 10^4^ cells/well) were seeded in 96-well plates and incubated overnight. Sera were heated at 56 °C for 30 min to inactivate complement and diluted in serum-free MEM at an initial dilution factor of 20, and then further twofold serial dilutions were performed for a total of 11 dilution steps to a final dilution of 1:40,960. The diluted sera were mixed with an equal volume of SARS-CoV-2 virus at 100 TCID_50_/50 μL (hCoV-19/Taiwan/CGMH-CGU-01/2020, GenBank accession MT192759) and incubated at 37 °C for 2 h. The sera-virus mixture was then added to 96-well plate with Vero E6 cells and incubated in MEM with 2% FBS at 37 °C for 5 days. After incubation, cells were fixed by adding 4% formalin to each of the wells for 10 min and stained with 0.1% crystal violet for visualization. Results were calculated with the Reed-Muench method for log 50% end point for ID_50_ and log 90% end point for ID_90_ titers.

### Ethical statements

Procedures involving wild-type SARS-CoV-2 virus followed the laboratory biosafety guidelines of the Taiwan CDC and were conducted in a biosafety level-3 facility at the Linkou Chang Gung Memorial Hospital. All animal works followed the guidelines in the Guide for the Care and Use of Laboratory Animals, National Academy Press (2010)^25^. All animal studies were reviewed and approved by the Institutional Animal Care and Use Committee (IACUC). The Testing Facility’s IACUC animal study protocol approval numbers are TFBS2020-006 and TFBS2020-010. All procedures involving study animals described in this study plan were conducted in a manner to avoid or minimize discomfort, distress or pain to the animals.

### Immunization of mice

Female BALB/c and C57BL/6 mice were obtained from the National Laboratory Animal Center, Academia Sinica, Taiwan and BioLASCO Taiwan Co. Ltd. For antigen formulation, SARS-CoV-2 S-2P protein was mixed with either an equal volume of CpG 1018, aluminum hydroxide, PBS, or CpG 1018 plus aluminum hydroxide. Mice aged 6–9 weeks were immunized twice (50 μL intramuscularly in each of the left and right quadriceps femoris muscles per mouse) at 3 weeks apart as previously described^8^. Total serum anti-S IgG and anti-RBD IgG titers were detected with direct ELISA using custom 96-well plates coated with S-2P antigen and an *E.* coli-expressed fragment of the S protein containing RBD region, respectively.

### Cytokine assays

Two weeks after the second injection, mice were euthanized and splenocytes were isolated and stimulated with S-2P protein (2 μg/well) as previously described^26^. For detection of IFN-γ, IL-2, IL-4, and IL-5, the culture supernatant from the 96-well microplates was harvested to analyze the levels of cytokines by ELISA using Mouse IFN-γ Quantikine ELISA Kit, Mouse IL-2 Quantikine ELISA Kit, Mouse IL-4 Quantikine ELISA Kit, and Mouse IL-5 Quantikine ELISA Kit (R&D System). The OD450 values were read by Multiskan GO (Thermo Fisher Scientific).

### Dose range finding study for single- and repeat-dose intramuscular injection (IM) in Sprague Dawley (SD) rats

Crl:CD Sprague Dawley (SD) rats were obtained from BioLASCO Taiwan Co. Ltd. Animal studies were conducted in the Testing Facility for Biological Safety, TFBS Bioscience Inc., Taiwan. SD rats aged 6–8 weeks were immunized with 5 μg, 25 μg or 50 μg of S-2P adjuvanted with either 1500 μg CpG 1018 alone or 750 μg CpG 1018 combined with 375 μg aluminum hydroxide. The test article or vehicle control was administered intramuscularly (0.25 mL/site, 2 sites of quadriceps femoris muscle) to each rat on Day 1 (for single-dose study) and Day 15 (for repeat-dose study). The observation period was 14 days (for single-dose study) and 28 days (for repeat-dose study). Parameters evaluated included clinical signs, local irritation examination, moribundity/mortality, body temperature, body weights, and food consumption during the in-life period. Blood samples were taken for hematology, including coagulation tests and serum chemistry. All animals were euthanized and necropsied for gross lesion examination, organ weights, and histopathology evaluation on injection sites and lungs.

### Statistical analysis

For neutralization assays, geometric mean titers are represented by the heights of bars with 95% confidence intervals represented by the error bars. For cytokine and rat data, heights of bars or symbols represent means with SD represented by error bars.

Dotted lines represent lower and upper limits of detection. Analysis package in Prism 6.01 (GraphPad) was used for statistical analysis. The data were compared at the same S-2P dose level with different adjuvant or at the same adjuvant system with varying antigen dose. Kruskal–Wallis with corrected Dunn’s multiple comparisons test was used for non-parametric test between more than 2 experimental groups. Mann–Whitney U-test was used to compare 2 experimental groups. For correlation between antibody titers and neutralization titers, Spearman’s rank correlation coefficient was used. *p < 0.05, **p < 0.01, ***p < 0.001.

## Supplementary information

Supplementary Information.

## Data Availability

The datasets generated during and/or analyzed during the current study are available from the corresponding author on reasonable request.

## References

[1] Zhou P (2020). A pneumonia outbreak associated with a new coronavirus of probable bat origin. Nature.

[2] Dong E, Du H, Gardner L (2020). An interactive web-based dashboard to track COVID-19 in real time. Lancet Inf. Dis..

[3] Gates B (2020). Responding to Covid-19—A once-in-a-century pandemic?. N. Engl. J. Med..

[4] Schäferhoff, M., Yamey, G. & McDade, K. Funding the development and manufacturing of COVID-19 vaccines: The need for global collective action. Brookings. https://www.brookings.edu/blog/future-development/2020/04/24/funding-the-development-and-manufacturing-of-covid-19-vaccines-the-need-for-global-collective-action/ Accessed 1 June 2020. (2020)

[5] WHO R&D Blueprint. DRAFT Landscape of COVID-19 Candidate Vaccines—31 July 2020. https://www.who.int/who-documents-detail/draft-landscape-of-covid-19-candidate-vaccines (2020)

[6] WHO R&D Blueprint. Target Product Profiles for COVID-19 Vaccines—17 April 2020. https://www.who.int/who-documents-detail/who-working-group-target-product-profiles-for-covid-19-vaccines (2020)

[7] Gorbalenya AE, Baker SC, Baric RS (2020). The species Severe acute respiratory syndrome-related coronavirus: Classifying 2019-nCoV and naming it SARS-CoV-2. Nat. Microbiol..

[8] Pallesen J (2017). Immunogenicity and structures of a rationally designed prefusion MERS-CoV spike antigen. Proc. Natl. Acad. Sci..

[9] Wrapp D (2020). Cryo-EM structure of the 2019-nCoV spike in the prefusion conformation. Science.

[10] Lee S, Nguyen MT (2015). Recent advances of vaccine adjuvants for infectious diseases. Immune Netw..

[11] Shi S, Zhu H, Xia X, Liang Z, Ma X, Sun B (2019). Vaccine adjuvants: Understanding the structure and mechanism of adjuvanticity. Vaccine..

[12] Tseng CT (2012). Immunization with SARS coronavirus vaccines leads to pulmonary immunopathology on challenge with the SARS virus. PLoS ONE.

[13] Hotez PJ, Corry DB, Bottazzi ME (2020). COVID-19 vaccine design: The Janus face of immune enhancement. Nat. Rev. Immunol..

[14] Campbell JD (2017). Development of the CpG adjuvant 1018: A case study. Methods Mol. Biol..

[15] Thomas LJ (2009). Co-administration of a CpG adjuvant (VaxImmune, CPG 7909) with CETP vaccines increased immunogenicity in rabbits and mice. Hum. Vaccin..

[16] Tian, J. H. *et al.* SARS-CoV-2 spike glycoprotein vaccine candidate NVX-CoV2373 elicits immunogenicity in baboons and protection in mice. *bioRxiv.*10.1101/2020.06.29.178509 (2020). 10.1038/s41467-020-20653-8 PMC7809486 33446655

[17] O’Flaherty R (2020). Mammalian cell culture for production of recombinant proteins: A review of the critical steps in their biomanufacturing. Biotechnol. Adv..

[18] Li, W. *et al.* Rapid selection of a human monoclonal antibody that potently neutralizes SARS-CoV-2 in two animal models. *bioRxiv*. 10.1101/2020.05.13.093088 (2020).

[19] Korber B (2020). Tracking changes in SARS-CoV-2 Spike: Evidence that D614G increases infectivity of the COVID-19 virus. Cell.

[20] Lopez AF, Begley CG, Williamson DJ, Warren DJ, Vadas MA, Sanderson CJ (1985). Murine eosinophil differentiation factor. An eosinophil-specific colony-stimulating factor with activity for human cells. J. Exp. Med..

[21] Jackson S (2017). Immunogenicity of a two-dose investigational hepatitis B vaccine, HBsAg-1018, using a toll-like receptor 9 agonist adjuvant compared with a licensed hepatitis B vaccine in adults. Vaccine..

[22] Hyer RN, Janssen RS (2019). Immunogenicity and safety of a 2-dose hepatitis B vaccine, HBsAg/CpG 1018, in persons with diabetes mellitus aged 60–70 years. Vaccine..

[23] Perazzo P (2015). Nanotechnology, drug delivery systems and their potential applications in hepatitis B vaccines. Int. J. Vaccines Vaccin..

[24] Huang CG (2020). Culture-based virus isolation to evaluate potential infectivity of clinical specimens tested for COVID-19. J. Clin. Microbiol..

[25] National Research Council (2010). Guide for the care and use of laboratory animals.

[26] Lu B (2010). Effect of mucosal and systemic immunization with virus-like particles of severe acute respiratory syndrome coronavirus in mice. Immunology.

