# Supplementary material for: Development of CpG-adjuvanted stable prefusion SARS-CoV-2 spike antigen as a subunit vaccine against COVID-19
Source: Sci Rep. 2020 Nov 18;10:20085. doi: 10.1038/s41598-020-77077-z (PMC7676267; doi:10.1038/s41598-020-77077-z)
Supplement: Supplementary file 1 — Supplementary Information. [file 41598_2020_77077_MOESM1_ESM.pdf]

Supplementary Information for:

Development of CpG-adjuvanted stable prefusion SARS-CoV-2 spike antigen as a subunit vaccine against COVID-19

Tsun-Yung Kuo[1,2], Meei-Yun Lin[1], Robert L Coffman[3], John D Campbell[3], Paula Traquina[3], Yi-Jiun Lin[1], Luke Tzu-Chi Liu[1], Jinyi Cheng[1], Yu-Chi Wu[1], Chung-Chin Wu[1], Wei-Hsuan Tang[1], Chung-Guei Huang[4,5], Kuo-Chien Tsao[4,5], Charles Chen[1,6*]

[1]Medigen Vaccine Biologics Corporation, Taipei City, Taiwan

[2]Department of Biotechnology and Animal Science, National Ilan University, Yilan County, Taiwan

[3]Dynavax Technologies, Emeryville, CA 94608, USA

[4]Department of Laboratory Medicine, Linkou Chang Gung Memorial Hospital, Taoyuan City, Taiwan

[5]Research Center for Emerging Viral Infections, College of Medicine, Chang Gung University, Taoyuan City, Taiwan

[6]Adjunct Professor of College of Science and Technology, Temple University, Philadelphia, PA 19122, USA

*Corresponding author: charles@medigenvac.com

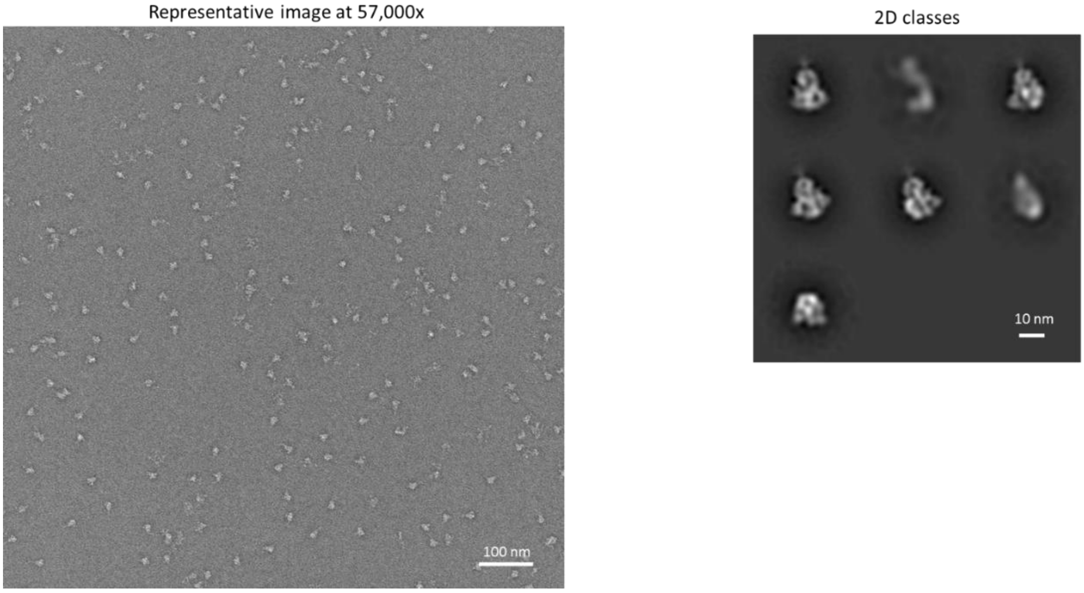

Supplementary Figure S1. Assembled spike trimers of SARS-CoV2 S-2P under EM (left) and corresponding to ordered spike molecules 2D classes (right). SARS-CoV2 S-2P was transiently expressed by ExpiCHO cells. The sample contains primarily assembled spike trimers. Most particles contributed to 2D classes corresponding to ordered spike molecules.

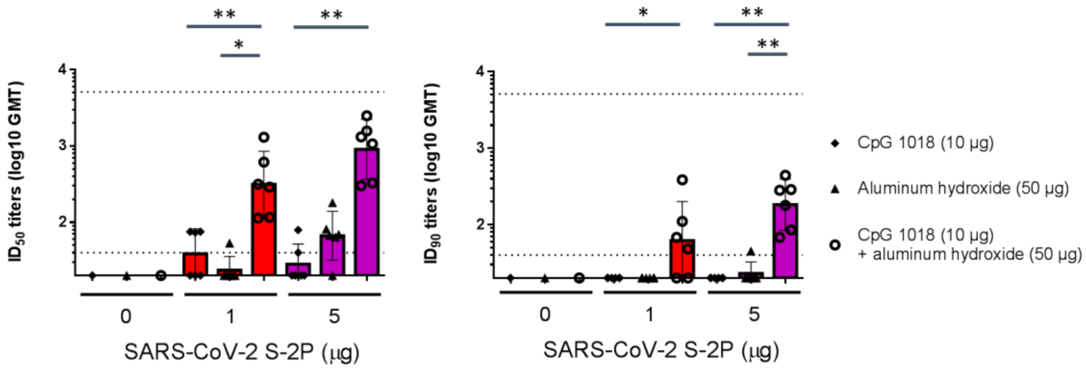

Supplementary Figure S2. Neutralizing antibody responses in BALB/c mice 3 weeks after first injection of CpG 1018 and aluminum hydroxide-adjuvanted SARS-CoV-2 S-2P. BALB/c mice (N=39) were immunized with 2 injections of CHO cell-expressed SARS-CoV-2 S-2P adjuvanted with CpG 1018, aluminum hydroxide or combination of both 3 weeks apart and the antisera were harvested at 3 weeks after the first injection. The antisera were subjected to neutralization assay with pseudovirus expressing SARS-CoV-2 spike protein to determine the ID_{50} (left) and ID_{90} (right) titers of neutralization antibodies.

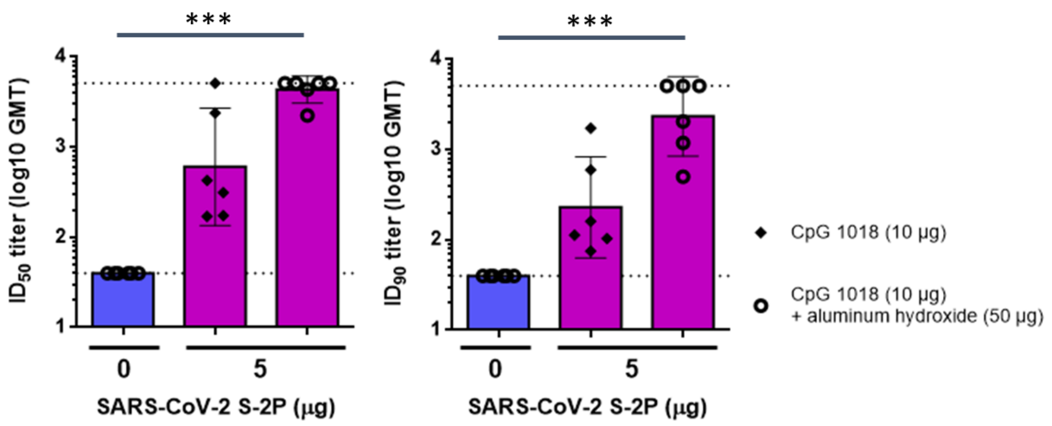

Supplementary Figure S3. Pseudovirus neutralization by antibodies induced by CpG 1018 and aluminum hydroxide-adjuvanted S-2P 2 weeks after the second injection. C57BL/6 mice (N=6 per group) were immunized with 0 or 5 μg of S-2P in the presence of CpG 1018 or CpG 1018 and alum. Sera were harvested for pseudovirus neutralization assay 2 weeks after the 2nd injection. ID_{50} (left) and ID_{90} (right) titers of neutralization antibodies were quantified.

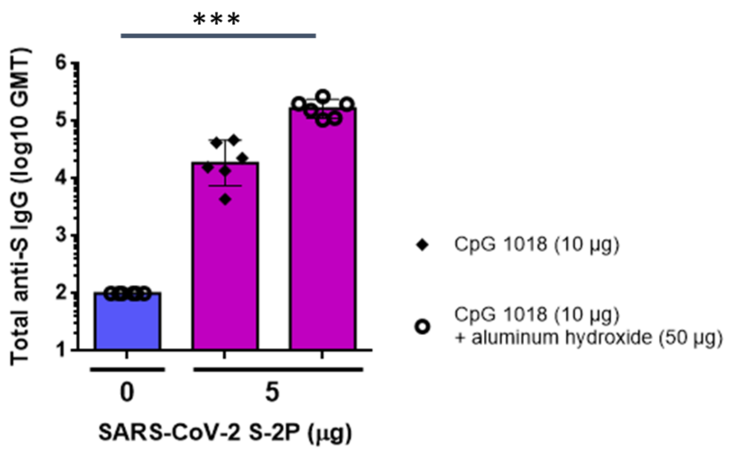

Supplementary Figure S4. Total anti-S IgG titers in C57BL/6 mice immunized with S-2P with adjuvants. C57BL/6 mice (N=6 per group) were immunized as in Supplementary Figure S3. Sera were harvested and quantified for the total amount of anti-S IgG with ELISA at 2 weeks after the 2nd injection.

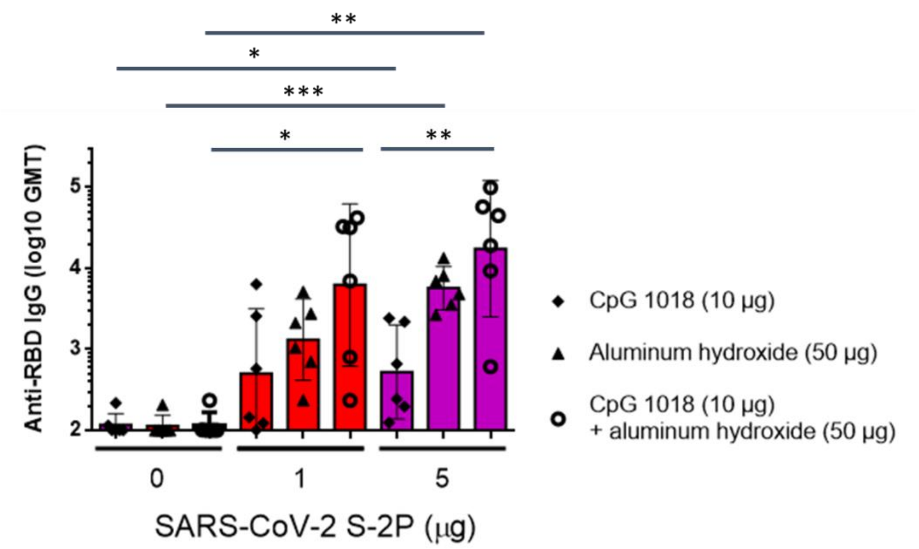

Supplementary Figure S5. Anti-RBD IgG titers in mice immunized with S-2P with ajuvants 2 weeks after the second injection. Sera from BALB/C mice as in Figure 2 (N=6 per group) were assayed for amount of anti-RBD IgG with ELISA coated with a fragment containing the RBD region of S protein.

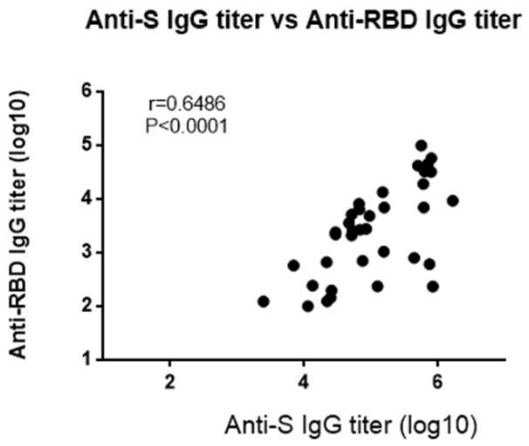

Supplementary Figure S6. Correlation between anti-S IgG titers and anti-RBD IgG titers. Values of anti-S IgG titers (Figure 2) and anti-RBD IgG titers (Figure S5) above lower detection limit (>40, N=36) were tabulated and Spearman's rank correlation coefficient was calculated.

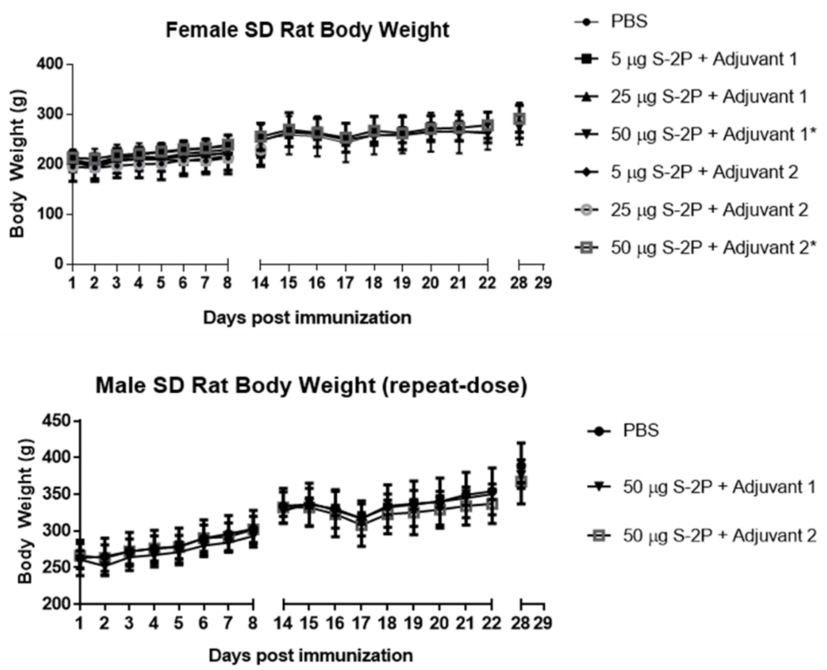

Supplementary Figure S7. Female (top, N=21) and male (bottom, N=9) body weight of SD rats immunized with indicated amount of S-2P with adjuvants. Adjuvant 1 = 1500 µg CpG 1018, Adjuvant 2 = 750 µg CpG 1018 + 375 µg aluminum hydroxide.

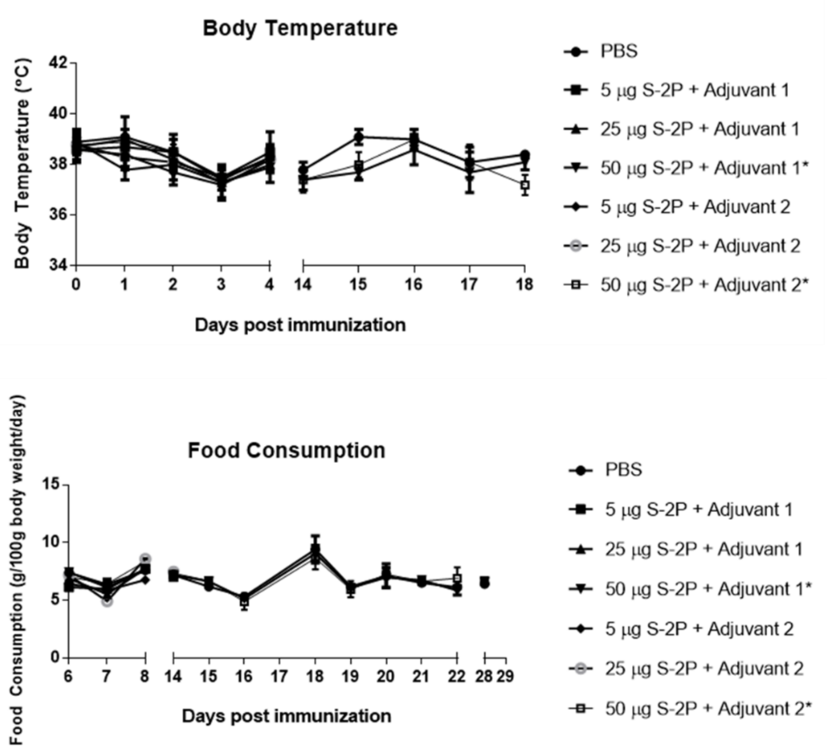

Supplementary Figure S8. Body temperature (top, N=30) and food consumption (bottom, N=10) of female and male SD rats immunized with indicated amount of S-2P with adjuvants. Adjuvant 1 = 1500 µg CpG 1018, Adjuvant 2 = 750 µg CpG 1018 + 375 µg aluminum hydroxide.
